# Epidemiological insights into carbapenem resistant infections in critical care settings: A molecular and clinical investigation

**DOI:** 10.2478/jccm-2025-0048

**Published:** 2025-10-31

**Authors:** Camelia Vintila, Razvan Lucian Coseriu, Alexandru Andrei Ujlaki Nagi, Adrian Man

**Affiliations:** George Emil Palade University of Medicine, Pharmacy, Science, and Technology of Targu Mures,Romania; Mures County Clinical Hospital, Targu Mures, Romania

**Keywords:** ERIC-PCR, AMR, MDR bacteria, ICU, bacterial screening

## Abstract

**Objective:**

This study aimed to investigate the prevalence and genetic relatedness of multidrug-resistant Gram-negative bacilli, particularly those resistant to carbapenems, in patients admitted to intensive care units. It also sought to explore associations between bacterial colonization or infection and clinical outcomes, including comorbidities, treatment regimens, and mortality.

**Methods:**

Between November 2022 and December 2023, screening and pathological samples were collected from patients at a tertiary hospital. Screening samples included rectal and pharyngeal swabs, while pathological samples comprised respiratory tract secretions. Bacterial identification and antibiotic susceptibility testing were performed using standard microbiological methods. Genetic similarity among isolates was assessed using a molecular fingerprinting technique to detect potential clonal spread.

**Results:**

A total of 62 carbapenem-resistant strains were identified, with Acinetobacter baumannii and Klebsiella pneumoniae being the most prevalent. Pathological isolates exhibited higher resistance levels than screening isolates. Most patients had multiple comorbidities, with cardiac, renal, and pulmonary conditions being the most common. A significant association was found between prolonged intensive care unit stay and increased mortality. However, no significant correlation was observed between the number of comorbidities or antibiotic classes used and mortality. Molecular analysis revealed clonal clusters of Acinetobacter and Klebsiella strains, suggesting nosocomial transmission.

**Conclusions:**

The findings underscore the importance of early screening, molecular surveillance, and stringent infection control measures in intensive care settings.

## Introduction

The discovery of antibiotics is undoubtedly of great importance to human health. Unfortunately, in the 21st century, overuse and inappropriate antibiotic consumption have driven the rapid development of multidrug-resistant (MDR) pathogens, making it difficult not only to limit their spread but especially to treat them [1]. MDR bacteria are bacterial strains that are resistant to multiple antibiotics, specifically to at least one antibiotic in three or more antimicrobial categories. Carbapenem resistance (CR) is critically important in the context of MDR bacteria because it often marks the last line of defense against severe infections, and its loss significantly limits treatment options, leading to higher morbidity and mortality. In a short period of time, the World Health Organization (WHO) has released statements referring to millions of deaths worldwide caused by MDR bacteria [2,3]. As a countermeasure, by November 2023, 178 countries had developed national antimicrobial resistance action plans, which include regulatory frameworks aimed at combating or limiting the spread of MDR bacteria [3].

In high-risk wards such as Oncology, Infectious Diseases, and especially Intensive Care Units (ICU), the screening of the patients for colonization by MDR organisms is a key measure. This allows for potential decolonization treatments, the use of prophylactic antibiotics before surgical interventions, and, most importantly, the isolation of patients, to prevent the transmission of these pathogens [4].

In ICUs, two major factors contributing to the emergence of antibiotic-resistant microorganisms were identified: antimicrobial selective pressure (caused by the use of broad-spectrum antibiotics, mainly carbapenems, which promotes the selection of MDR bacteria), and the transmission of resistant organisms by healthcare personnel [5]. Moreover, the growing number of invasive procedures in ICU contributes to the spread of bacteria into normally sterile areas of the body, leading to increased chance to develop lower respiratory tract infections (LRTI) including ventilator-associated pneumonia, surgical site infections, catheter-related bloodstream or urinary tract infections [6]. From an epidemiological perspective, tracking the prevalence and persistence of the micro-organisms involved in these infections is essential due to the severe outcome they can cause, which often lead to death. For this reason, it is essential to make use of molecular tests such as ERIC-PCR to accurately detect and confirm potential intrahospital transmission of MDR bacteria [7], or to determine whether an initial colonizer has contributed to the development of a LRTI during hospitalization.

The study aims to explore the epidemiology of multidrug-resistant Gram-negative bacilli, with particular emphasis on carbapenem resistance, in high-risk hospital wards - particularly the ICU - based on screening tests conducted on pathological samples collected both prior to admission and during hospitalization. Additionally, it investigates potential correlations between the duration of hospitalization, administered treatments, comorbidities, mortality, and the isolated pathogens. Furthermore, molecular testing methods appreciated the possible clonal spread of MDR Gram-negative bacilli for epidemiological analysis.

## Methods

The study was conducted between November 2022 and December 2023, using samples collected at Mureș Clinical County Hospital (MCCH) with the approval of Ethical Board (no. 2618 from April 2024).

Samples were collected from ICU patients and divided into two categories: (1) screening samples for CR bacteria, and (2) respiratory specimens. All age groups were included. Only the first bacterial isolate per patient was analyzed to exclude duplicates and chronic colonization. Selection was limited to bacterial strains exhibiting both multidrug resistance and carbapenem resistance.

Screening samples consisted of rectal and pharyngeal swabs obtained either at admission or during hospitalization. Respiratory specimens - including tracheal and bronchial aspirates, sputum, and intra-/orotracheal cannula aspirates - were collected in the clinical context of suspected lower respiratory tract infection and yielded CR isolates.

### Samples selection and patient data

Screening samples were processed in accordance with the laboratory’s internal protocols and inoculated on CRE Brilliance Agar (Oxoid, Hampshire, UK), then incubated under atmospheric conditions at 35°C for 18–24 hours. The growth of bacterial colonies presumptively indicated the presence of potential carbapenem-resistant Gram-negative glucose-fermentative bacilli (such as *Klebsiella* spp., *Escherichia coli*, *Serratia* spp., *Providencia* spp., etc) or non-glucose-fermentative bacilli (*Pseudomonas* spp., *Acinetobacter* spp., *Stenotrophomonas* spp., *Burkholderia* spp., etc).

Pathological samples were processed following the routine microbiological laboratory procedures, being inoculated on Columbia Agar (Oxoid, Hampshire, UK) and CLED Agar (Cystine Lactose Electrolyte-Deficient; Oxoid, Hampshire, UK), and incubated at 35°C for 18–24 hours. The suggestive bacterial colonies were isolated, identified based on phenotypic and biochemical characteristics or using the automated Vitek 2 Compact system (bioMérieux, Marcy-l’Étoile, France), and tested for their antibiotic susceptibility.

All strains (both from screening and pathological products) were tested for their antibiotic susceptibility using the Kirby-Bauer disk diffusion method on Mueller-Hinton Agar (Oxoid, Hampshire, UK), as well as by determining the minimum inhibitory concentration (MIC) with the Vitek 2 system, following the EUCAST 2022 (version 12) and 2023 (version 13.1) (European Committee on Antimicrobial Susceptibility Testing) guidelines[8]. Colistin was tested only for clinical isolates. Strains that were resistant to meropenem were considered carbapenem resistant (CR) and stored at −70°C for further testing.

For all patients that proved to be colonized or infected with MDR Gram-negative bacteria, clinical data was retrieved from the hospital informatic system: ward of admission, prior infections and treatments, ward transfers, comorbidities, age, sex, and other relevant clinical details.

### Genetic analysis

To investigate genetic similarity, the Enterobacterial Repetitive Intergenic Consensus Polymerase Chain Reaction (ERIC-PCR) method was employed to detect potential clonal spread of carbapenem-resistant bacterial strains [9]. This technique amplifies multiple palindromic ERIC sequences dispersed throughout the bacterial genome, using specific primers that target conserved regions. The resulting amplification products vary in molecular size, generating a unique electrophoretic pattern for each bacterial strain.

To obtain pure and high-quality DNA, the IndiSpin Pathogen Kit (Indical Bioscience, Leipzig, Germany) was used according to the manufacturer’s protocol. A final volume of 50 µL of DNA was obtained and stored at −20°C for further analysis.

For ERIC-PCR, the reaction mixture consisted of 12.5 µL of DreamTaq Green PCR Master Mix 2X (Thermo Fisher Scientific, Waltham, MA, USA), 0.4 mM of forward ERIC primer (5′-ATGTAAGCTCCTGGGGATTCAC-3′), 0.4 mM of reverse ERIC primer (5′-AAGTAAGTGACTGGGGTGAGCG-3′), 1 µL of DNA template, and DNase-free water to a final volume of 25 µL.

The amplification protocol included an initial denaturation at 95°C for 5 minutes, followed by 30 cycles of denaturation at 94°C for 30 seconds, primers annealing at 52°C for 1 minute, and elongation at 72°C for 2 minutes. A final elongation step was performed at 72°C for 8 minutes.

The final results were visualized by adding 10 µL of amplification product in 2% electrophoresis agarose gel, prepared using Grade Electran® DNA Agarose and Tris-Borate-EDTA (TBE) Buffer. For UV visualization of DNA, one microliter of RedSafe® (Sigma-Adrich, Saint Louis, Missouri, USA) was used for gel staining. The first lane of each electrophoresis gel was loaded with 1 µL GeneRuler 100 bp DNA Ladder (Thermo Fisher Scientific, Waltham, MA USA).

The electrophoresis was run in 1X TBE Buffer at 65 Volts (5 volts/centimeter) for 2.5 hours. The ERIC-PCR dendrogram was created using GelJ Software (UPGMA method with 1% band-matching tolerance, with band matching tolerance established at 1) based on the images captured with the MiniBIS Pro imaging device (Bio-Imaging Systems, Modi’in-Maccabim-Re’ut, Israel). The strains that presented at least 80% pattern similarity were considered highly related, while a similarity of 100% would prove clonality.

### Statistical analysis

The collected data were entered and organized using Microsoft Excel 2019. Statistical analyses were performed using GraphPad InStat 3 software (GraphPad Software, San Diego, CA, USA). Categorical variables were compared using Fisher’s exact test. Associations between binary variables were assessed by calculating odds ratios (OR) along with 95% confidence intervals (95% CI). A p-value of less than 0.05 was considered statistically significant.

## Results

After applying the inclusion criteria, 62 strains were selected. Of these, 32 CR strains were identified from screening samples. *Acinetobacter baumannii* accounted for the majority (n = 12; 37.5%), followed by *Pseudomonas aeruginosa* (n = 8; 25%), *Stenotrophomonas maltophilia*, and *Klebsiella pneumoniae* (each n = 6; 18.75%). From pathological samples, 30 strains were identified as CR and selected for further analysis. Among these, *K. pneumoniae* was the most prevalent (n = 14; 46.66%), followed by *A. baumannii* (n = 12; 40%) and *P. aeruginosa* (n = 4; 13.33%). None of the strains isolated from pharyngeal swabs were recovered from lower respiratory tract samples, indicating a lack of clonal relatedness between isolates from the two sites.

### Bacterial strains and patients

**Fig. 1.**
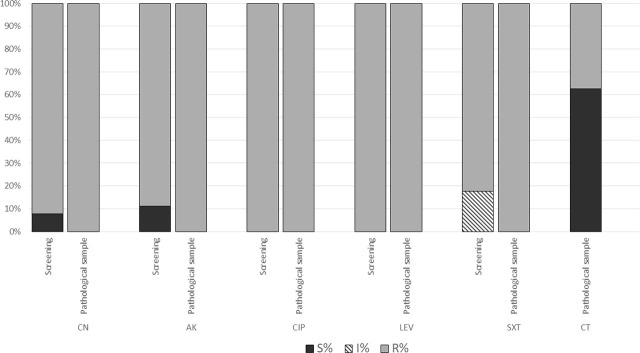
Comparative resistance of microbial strains

The CR samples that were isolated either from screening or from pathological products presented multiple associated resistance levels to other antibiotic classes, as presented in Figure 1.

The strains isolated from pathological samples showed high resistance to aminoglycosides and fluoroquinolones (n = 30; 100%), with 37% also exhibiting resistance to colistin. The CR strains from screening samples demonstrated the same resistance trend to all antibiotic classes, excepting aminoglycosides and trimethoprim-sulfamethoxazole where susceptible strains were present (<10%).

**Fig. 2.**
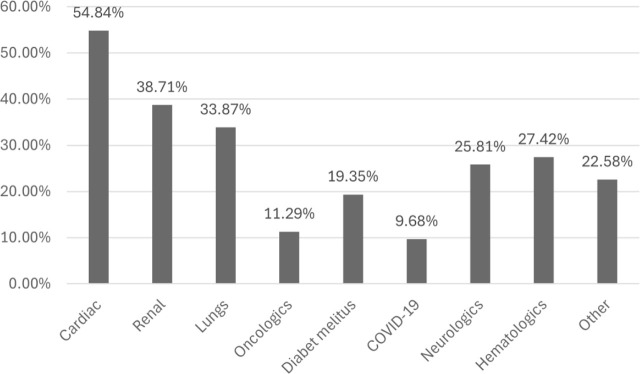
The percent of patients presenting comorbidities

Patients admitted to the ICU presented with multiple comorbidities (Figure 2), the most common being cardiac conditions (n = 33; 54.84%), followed by renal (n = 21; 38.71%) and pulmonary diseases (n = 20; 33.87%). Other conditions included oncological diseases, diabetes, and in the less amount COVID-19. The comorbidities were systematically categorized, revealing that the majority of patients presented with more than three conditions across multiple diagnostic categories. Among these, cardiovascular disorders emerged as the most prevalent.

Statistical analysis indicating no significant correlation between the number of comorbidities and patient mortality (p-value 0.1, CI 95%) (for both the cases with positive results for MDR screening or from pathological samples). Similar non-significant results were observed when analyzing the relationship between ICU hospitalization frequency and the presence of comorbidities.

The patients included in the study had an average age of 67.01 years (SD=17.85) and were predominantly males (n=51; 82.25%) as presented in Table 1 and 2.

**Table 1.** Demographic and clinical characteristics of data collected from patients with screening samples by age and sex

**Screening samples**			**Comorbidities**			**Number of patients with susceptible results to different antibiotic classes, other than beta-lactamins**			

**Age**	**Sex**		**0**	**1**	**≥2**	**AG**	**FQ**	**SXT**	**Deceased**
<18	Male	n= 2 6.25%	n=2 100%	-	-	-	-	-	-
	Female	n= 1 3.12%	n=1 100%	-	-	-	-	-	-

19–60	Male	n= 4 18.75%	-	n=1 16.66%	n=3 83.33%	n= 1	n= 1	-	n=4 100%
	Female	n =2 6.25%	-	-	n=2 100%	n=1	n=1	-	n=0

>61	Male	n= 20 62.5%	n=3 15%	n=5 25%	n=12 60%	-	-	-	n= 15 75%
	Female	n= 3 9.37%	-	n=1 33.3%	n=2 66.66%	-	-	-	n= 2 66.66%

AG- aminoglycosides, FQ- fluoroquinolones, SXT- Trimethoprim with sulfamethoxazole

**Table 2.** Demographic and clinical characteristics of data collected from patients with LRT pathological samples by age and sex

**Pathological samples**			**Comorbidities**			**number of patients with susceptible results to different antibiotic classes, other than beta-lactamins**				

**Age**	**Sex**		**0**	**1**	**≥2**	**AG**	**FQ**	**SXT**	**CT**	**Decesed**
<18	Male	n= 1 3.33%	n=1 100%	-		-	-	-	-	-
	Female	n= 0	-	-	-	-	-	-	-	-

19–60	Male	n= 4 13.33%	-	n=4 100%	-	-	-	-	n=3 75%	n=3 75%
	Female	n =0	-	-	-	-	-	-	-	n=0

>61	Male	n= 20 66.6%	n=2 10%	n=3 15%	n=15 75%	-	-	-	n=7 35%	n= 16 80%
	Female	n= 5 16.66%	n=1 20%	n=1 20%	n=3 60%	-	-	-	n=4 75%	n= 3 60%

AG- aminoglycosides, FQ- fluoroquinolones, SXT- Trimethoprim with sulfamethoxazole, CT-Colistin

The patients were initially hospitalized in various wards, but due to the deterioration of their clinical health, they required transfer to the ICU. The average total duration of hospitalization was 18.96 days (ranging from 4 days to 121 days), while the average ICU stay was 8.88 days (ranging from a few hours to a maximum of 118 days). Notably, 70.96% of the patients in our study group who were admitted to the ICU did not survive.

To investigate the correlation between ICU, stay duration and mortality, patients were divided into two groups: those with an ICU stay of fewer than 7 days and those with a stay of 8 days or more. A statistically significant association was observed between prolonged ICU stay and mortality (p = 0.0002), with an odds ratio (OR) of 18.3 (95% CI). This indicates that patients hospitalized in the ICU for 8 days or more had an approximately 18-fold higher risk of death compared to those with shorter ICU stays. These findings highlight that the duration of ICU stay is critical factor influencing patient outcomes.

Additionally, the study investigated the relationship between antibiotic use and mortality. Patients were categorized into two groups: those receiving moderate treatment (a maximum of two antibiotics from different classes) and those receiving excessive treatment (three or more antibiotics from different classes). Although some patients received treatment involving 8 to 10 antibiotic classes in a relatively short period (72% received colistin, 34% vancomycin, and 30% both antibiotics), statistical analysis revealed no significant association between excessive antibiotic use and mortality (p = 0.06).

### Molecular results

**Fig. 3.**
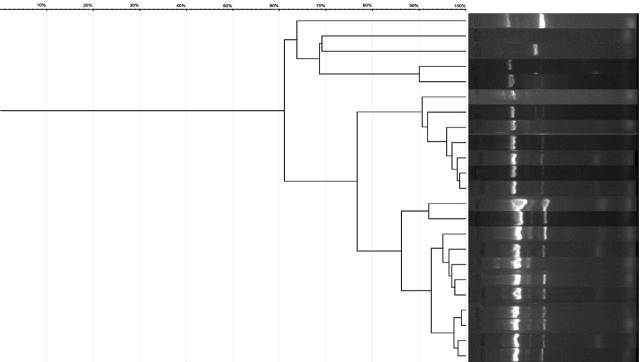
**ERIC-PCR patterns of** *A. baumannii* **isolates**

The possible clonal spreading of bacteria in the ICU hospitalized patients was assessed by ERIC-PCR, the results being presented in Figure 3.

According to the ERIC-PCR results, two main clusters of *Acinetobacter* spp. were identified, consisting of 11 respectively 7 strains with high genetic similarity (90–98%), indicating they are highly related. After thorough analysis, we concluded that the strains in the first cluster—comprising 7 *Acinetobacter* spp. isolates—were admitted to the ICU between January 27, 2023, and February 7, 2023, with one exception on April 3, 2023. In contrast, no clear temporal pattern could be established for the second cluster. Although it also showed high similarity, the ICU admission dates for these strains ranged from January 20, 2023, to August 4, 2023. Notably, four strains within this cluster, which exhibited 97–99% similarity, were admitted between January 20, 2023, and February 1, 2023.

**Fig. 4.**
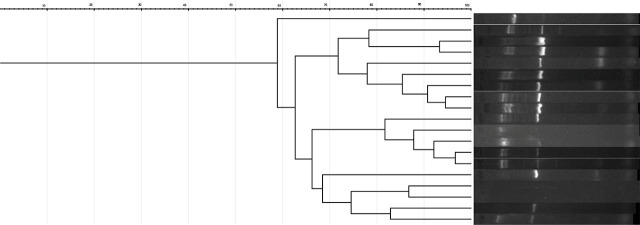
**ERIC-PCR patterns of** *Klebsiella* **spp. isolates**

For *Klebsiella* (Figure 4), one cluster was able to be identified, with similarities between 80 and 97%, composed of 5 strains of *K. pneumoniae*. Three of the patients were admitted to ICU in the period 08.2023 and 11.2023, and two of them (Sample 13 and 14) on the same day. The rest of the samples with similarity under 80% were considered unidentical.

**Fig. 5.**
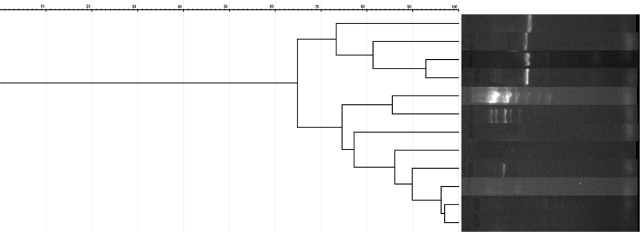
**ERIC-PCR patterns of** *P. aeruginosa* **isolates**

No identical clones were identified in the case of *Pseudomonas* strains (Figure 5), their similarity exceeding 80% only in isolated cases. Four strains showed a similarity of over 97%, having a relatively close hospitalization period, between 08.2023 and 11.2023.

**Fig. 6.**
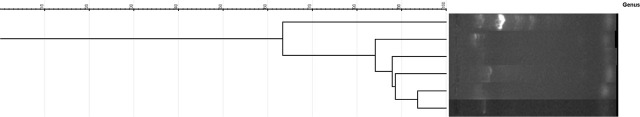
ERIC-PCR patterns of *S. maltophilia* isolates

In the case of *Stenotrophomonas* (Figure 6), 5 strains out of the 6 identified had a similarity of over 80% and were considered highly related. Upon closer investigation, it was determined that 4 out of the 6 strains had initially different admissions wards (Plastic Surgery, Oncology and Radiology) and were subsequently transferred to the ICU. Two of them had the same admission date in the ICU. The other 2 strains of *Stenotrophomonas* were collected from screening in the neonatology ICU from twins.

## Discussions

The identification of potential multidrug-resistant (MDR) bacteria in high-risk patients is widely recognized in scientific literature as a critical surveillance tool. More than a decade has passed since the European Centre for Disease Prevention and Control (ECDC) emphasized the importance of conducting such screenings to detect bacteria with various resistance mechanisms. In Romania, this practice has since become mandatory through legislative measures [10]. The results of screening, as reported in the literature, are often ambiguous. In Belgium, a study by Abramowicz et al. reported a 41% colonization rate with MDR Gram-negative bacteria. The distribution of bacterial species isolated in our study, based on the inclusion criteria, revealed a high prevalence of *A. baumannii*, which aligns with existing literature highlighting its frequent involvement in nosocomial infections, particularly in intensive care units.

In contrast, *K. pneumoniae* was the most frequently isolated species from pathological samples (46.66%). This inversion in proportions suggests a potential difference in bacterial tropism or infection source, possibly reflecting specific clinical contexts or characteristics of the studied population. Moreover, the consistent presence of *A. baumannii* in both screening and pathological samples underscores its significance as a major opportunistic pathogen with a high potential for antibiotic resistance [11]. In contrast to a study conducted in Finland, where 59% of the 231 CPE strains were identified through screening [12], our data show a more balanced distribution, with a significantly lower proportion of positive screening samples during the period November 2022 to December 2023 (n = 37; 6.95%).

The trend we can observe is an increase, the number of CPE isolated from screenings increases in relatively short periods of time. A study conducted in the UK (United Kingdom) shows an increase from 2651 isolates in 2013 to 10589 in 2015. However, what we can observe is the fact that, even though 87% of the strains were isolated from screening, not all of them caused infections. The same thing can be observed in the case of our study, there being a very small number of patients who presented a positive screening, and the same species was also identified from a pathological product [13]. Statistical analysis revealed a significant association between prolonged ICU stay and increased mortality (OR = 18.3; p = 0.0002). Patients hospitalized for 8 days or more had a markedly higher risk of death compared to those with shorter ICU stays. However, given the observational nature of the study, this association should be interpreted with caution. Potential confounding factors—such as underlying illness severity, clinical indication for prolonged care, and time-dependent biases (e.g., immortal time bias)—may have influenced the observed relationship. These aspects warrant further investigation in controlled or longitudinal studies. The detection of clonal bacterial strains highlights the need for enhanced epidemiological monitoring to prevent the spread of multidrug-resistant pathogens.

Several studies have been conducted since the 1990s, including one by the European Prevalence of Infections in Intensive Care Units (EPIC) focused on Eastern Europe. This study revealed that 42% of patients were suspected of or confirmed to have infections. Alarmingly, 62% of these patients were already receiving antibiotics, whether prophylactic or therapeutic. Years later, in 2007, another study was carried out similarly, resulting in nearly identical findings, with 51% of patients affected. What is surprising is that the prescription of antibiotics was even bigger 71% [14]. The high level of antibiotic resistance observed among strains isolated from pathological specimens is alarming, as all 30 CR isolates also exhibited high resistance to aminoglycosides and fluoroquinolones, with 37% additionally resistant to colistin - an antibiotic of last resort. These findings indicate substantial selective pressure within the intensive care environment, where extensive antibiotic use may promote the emergence of multidrug-resistant strains. In comparison, strains isolated from screening samples collected prior to ICU admission demonstrated lower resistance levels, with no antibiotic class showing 100% resistance. This contrast underscores the impact of the hospital environment on bacterial selection and highlights the urgent need for stringent infection control measures and prudent antibiotic stewardship. Regarding patient profiles, the majority of ICU-admitted individuals presented with multiple comorbidities, the most common being cardiac (54.84%), renal (38.71%), and pulmonary conditions (33.87%). Unfortunately, our study reveals that the same practices regarding the administration of prophylactic antibiotics have been maintained, even from the first day of admission to the ICU. Some of the patients were receiving more than five different classes of antibiotics in a short span of time. Although our statistical analysis did not demonstrate a significant association between the number of antibiotic classes administered and mortality (p = 0.06, CI 95%), we observed a trend toward worse outcomes in patients receiving multiple antibiotics. This trend may reflect underlying clinical severity or other confounding factors rather than a direct causal relationship. While broad-spectrum antibiotics can be beneficial in critical care settings, their extensive use may contribute to the selection of resistant strains, potentially complicating treatment outcomes. These findings highlight the need for further investigation through adjusted analyses and underscore the importance of antibiotic stewardship. In 2017 a large study was conducted by Campion et al., including Romania, 70% were receiving at least one class of antibiotic, and 28% receiving prophylactic antibiotics in most cases cephalosporines. Improper or inefficient use of antibiotics can result in prolonged hospital stays, the emergence of multidrug-resistant infections, and increased mortality rates. Patients in critical condition in intensive care, especially those experiencing severe sepsis and septic shock, are susceptible to antibiotic treatment failures and secondary infections linked to inappropriate antibiotic use [15].

The presence of multidrug-resistant (MDR) bacteria among patients in intensive care units (ICUs) not only jeopardizes their health but also facilitates the acquisition of plasmids containing resistance genes, which play a significant role in the adaptation and dissemination of bacteria under adverse conditions. Despite the implementation of measures aimed at curbing the spread of MDR bacteria, instances of isolating the same bacterial species consecutively from ICU patients are relatively common. Consequently, these strains have undergone further investigation through ERIC-PCR, which has demonstrated genetic similarity among them. Similarly, other studies have reported comparable findings, revealing clusters of high genetic similarity among *A. baumannii* isolates [14].

Although ERIC-PCR provides a rapid and cost-effective method for assessing genetic relatedness, its discriminatory power is lower than that of PFGE or WGS, which may limit the precision of clonal relationship assessments. Nevertheless, there are studies that support the epidemiological role of ERIC-PCR, especially given the low costs and low complexity of the method [16,17]. The ERIC-PCR analysis of CR isolates revealed clusters of highly related strains (≥80% similarity), with a subset showing 100% similarity, which we defined as clonal. These findings suggest a potential recent transmission event or common source within the ICU. To ensure terminological consistency, we have reserved the term “identical clones” exclusively for strains with 100% similarity, while strains with 90–98% similarity are described as “closely related” or “highly related,” in accordance with our methodological thresholds. In contrast, other isolates showed high genetic diversity, indicating multiple introductions or widespread circulation of distinct clones in the hospital environment. These findings highlight both clonal spread and diverse sources contributing to CR infections. The results also underscore the importance of molecular typing in identifying sources and transmission routes of nosocomial infections, as well as the need for rigorous infection control measures to prevent the spread of these multidrug-resistant strains.

## Conclusion

The study highlights the critical role of early screening and molecular surveillance in detecting and controlling the spread of multidrug-resistant bacteria in ICU settings. This study emphasizes the urgent need for a proactive and integrated approach to antimicrobial resistance in critical care settings. Beyond isolated interventions, sustainable progress depends on coordinated surveillance, responsible antibiotic use, and rapid response to emerging threats. Strengthening these pillars is essential to protect vulnerable patients and preserve the effectiveness of life-saving treatments. These findings underscore the urgent need for targeted infection control strategies and rational antibiotic stewardship to mitigate the impact of MDR pathogens.
